# Leukocyte metabolism in obese type 2 diabetic individuals associated with COVID-19 severity

**DOI:** 10.3389/fmicb.2022.1037469

**Published:** 2022-11-03

**Authors:** Tiago Bertola Lobato, Matheus Gennari-Felipe, Janaína Ribeiro Barbosa Pauferro, Ilana Souza Correa, Beatriz Ferreira Santos, Beatriz Belmiro Dias, João Carlos de Oliveira Borges, Camila Soares dos Santos, Elvirah Samantha de Sousa Santos, Maria Janaína Leite de Araújo, Liliane Araújo Ferreira, Sara Araujo Pereira, Tamires Duarte Afonso Serdan, Adriana Cristina Levada-Pires, Elaine Hatanaka, Leandro Borges, Maria Fernanda Cury-Boaventura, Marco Aurélio Ramirez Vinolo, Tania Cristina Pithon-Curi, Laureane Nunes Masi, Rui Curi, Sandro Massao Hirabara, Renata Gorjão

**Affiliations:** ^1^Programa de Pós-graduação Interdisciplinar em Ciências da Saúde, Universidade Cruzeiro do Sul, São Paulo, São Paulo, Brasil; ^2^Department of Molecular Pathobiology, New York University, New York, NY, United States; ^3^Laboratory of Immunoinflammation, Department of Genetics, Evolution, Microbiology, and Immunology, Institute of Biology, University of Campinas, Campinas, Brazil; ^4^Immunobiological Production Section, Bioindustrial Center, Butantan Institute, São Paulo, Brazil

**Keywords:** SARS-COV-2, lymphocyte, macrophage, neutrophil, insulin resistance

## Abstract

Recent studies show that the metabolic characteristics of different leukocytes, such as, lymphocytes, neutrophils, and macrophages, undergo changes both in the face of infection with SARS-CoV-2 and in obesity and type 2 diabetes mellitus (DM2) condition. Thus, the objective of this review is to establish a correlation between the metabolic changes caused in leukocytes in DM2 and obesity that may favor a worse prognosis during SARS-Cov-2 infection. Chronic inflammation and hyperglycemia, specific and usual characteristics of obesity and DM2, contributes for the SARS-CoV-2 replication and metabolic disturbances in different leukocytes, favoring the proinflammatory response of these cells. Thus, obesity and DM2 are important risk factors for pro-inflammatory response and metabolic dysregulation that can favor the occurrence of the cytokine storm, implicated in the severity and high mortality risk of the COVID-19 in these patients.

## Introduction

The coronavirus belongs to the RNA virus family, widely distributed among mammals and birds, mainly causing respiratory or enteric diseases. In some cases, it can also cause liver and neurological diseases (Cui et al., 2019; Carod Artal, 2020). Different coronavirus strains infect their hosts in a specific way; these infections can be acute or prolonged. The main routes of virus transmission include respiratory and fecal-oral pathways. Coronaviruses present the largest genome among all RNA viruses, a specific characteristic of this family (Masters, 2006).

Two different highly pathogenic coronaviruses were responsible for two of the major viral epidemics in the last two decades: the Severe Acute Respiratory Syndrome Coronavirus (SARS-CoV), which originated in China in 2002–2003, and the Middle East Respiratory Syndrome Coronavirus (MERS-CoV), which originated in the Middle East in 2012 (Zhu et al., 2020; Zhou et al., 2020a). Both coronaviruses have a zoonotic origin and the ability to cause severe and fatal diseases in humans (Andersen et al., 2020; Zhu et al., 2020). At the end of December 2019, the outbreak of a new coronavirus in Wuhan, located in Hubei province in China, was described in several patients showing similar clinical symptoms, including fever, cough, dyspnea, and atypical pneumonia (Zhou et al., 2020a). The pathogen was identified in bronchoalveolar lavage fluid from a patient with “pneumonia of unknown etiology” after sequencing the virus genome. Bioinformatic analyses revealed that the virus’s characteristics were typical of the beta-coronavirus 2B type strain (WHO, 2020). In addition, a 96% similarity was identified with the genome of the bat SARS-like coronavirus of the BatCov strain RaTG13, a coronavirus detected in the species *Rhinolophus affinis*, from Yunan province in China (Zhou et al., 2020a).

The virus was initially named 2019-novel coronavirus (2019-nCoV). Subsequently, on February 11, 2020, the World Health Organization (WHO) altered the name to SARS-CoV-2 due to its close resemblance to the SARS-CoV (Velavan and Meyer, 2020). In January 2020, the virus was already manifesting outside China. The WHO declared a global health emergency on January 30, 2020, and the COVID-19 pandemic on March 11, 2020 (WHO, 2020).

SARS-CoV-2 is a ribonucleic acid (RNA) virus with about 30,000 nucleotides and 29 translated viral proteins (Ceraolo and Giorgi, 2020; Di Mascio et al., 2021). The spike glycoprotein (*S* protein) is responsible for the entry of the virus into the host cell through binding to the ACE-2 receptor and subsequent fusion to the plasma membrane. Like other coronaviruses, SARS-CoV-2 needs proteolytic processing of *S* protein to activate the endocytic pathway. Host proteases participate in this cleavage and activation of the SARS-CoV-2 for entering into the cell; among these are the transmembrane protease serine-2 (TMPRSS-2), cathepsin L, and furin (Hoffmann et al., 2020; Shang et al., 2020). Single-cell RNA sequencing data showed that TMPRSS2 is highly expressed in different tissues with co-expression of ACE-2, including epithelial cells in the nose, lungs, and bronchial branches, which explains, at least in part, the tissue tropism for the SARS-CoV-2 (Lukassen et al., 2020; Sungnak et al., 2020). Another essential viral protein is the nucleocapsid (*N* protein), which regulates the viral replication process in the host cell (Luo, 2012). Since SARS-CoV-2 is a RNA virus, it can be directly translated by the host cell machinery to produce viral proteins (Khan et al., 2021).

In response to the SARS-CoV-2 infection, the host organism activates its defense systems by increasing immune cell-mediated inflammatory processes. Immune and epithelial cells produce and release several cytokines with a pro-inflammatory profile, which leads to cytokine storm when released in a persistent and/or exacerbated way, thus causing an uncontrolled inflammatory response and severe symptoms in the patients. Individuals with comorbidities, including obesity, hypertension, type 2 diabetes mellitus, cancer, and autoimmune diseases, as well elderly individuals, have a high risk of presenting exacerbated inflammatory response associated with COVID-19, resulting in a poor prognosis and high mortality rate (Wang et al., 2019; Bolay et al., 2020; Cai et al., 2020; Wilk et al., 2020; Xu et al., 2020; Zhou et al., 2020a). Recent studies show that the metabolic characteristics of different leukocytes undergo changes in face of infection with SARS-CoV-2 infection and in obese type 2 diabetic individuals.

## Metabolic changes in leukocytes

### Neutrophils

Neutrophils are one of the first inflammatory leukocytes recruited to places of host damage. These leukocytes have long been viewed as short-lived essential cells for the elimination of extracellular pathogens, possessing a restricted function in the orchestration of the inflammatory response and immune function. However, they can also move away from injured tissues, what is called reverse migration, having effects on other cells thus exerting beneficial or harmful effects depending on the context (Ji and Fan, 2021). In addition, neutrophils also directly and indirectly (i.e., through inflammatory mediators) interact with macrophages, dendritic cells, and lymphocyte subsets present at inflammatory sites and regulate their effector functions. Neutrophils have emerged as a significant source of humoral pattern recognition molecules that identify pathogen-associated molecular patterns (PAMPs) and start the immune response in coordination with the cellular arm, thus responding as functional ancestors of antibodies (Mantovani et al., 2011).

As part of the innate immune response, neutrophils have four primary responses, including phagocytosis, degranulation, production of reactive oxygen species (ROS), and neutrophil extracellular traps (NETs) formation (van der Linden and Meyaard, 2016).

NETosis represents an important function of neutrophils to create and extrude complexes of decondensed DNA, termed NETs (Brinkmann et al., 2004). The NETs play a protective role in the immune system against invading pathogens. Also, they possess pro-inflammatory properties that can induce coagulation and thrombosis (Martinod and Wagner, 2014). Neutrophils regulate acute inflammation and the subsequent infiltration of other immune cells through enzymes, such as neutrophil elastase (NE) or myeloperoxidase (MPO), and antimicrobial peptides, as well as by secreting chemokines and pro-inflammatory cytokines (Mantovani et al., 2011).

The NETs are formed by neutrophil’s nuclear DNA fibers released in the extracellular space in response to acute and chronic inflammation, infection, and activated platelets. The generation of NETs is a controlled response by NETosis, a specific type of cell death different from apoptosis and necrosis. In general, the NETosis process is dependent on the production of ROS by NADPH oxidase (Delgado-Rizo et al., 2017) and includes the release of nuclear chromatin lined with effector proteins and peptidyl arginine deiminase type IV (PAD4) activation (Yousefi et al., 2019). After stimulation, the neutrophil nuclear envelope disintegrates to enable the mixing of chromatin with granular proteins (Fuchs et al., 2007). MPO and NE promote chromatin condensation and deteriorate histones (Papayannopoulos et al., 2010). In the presence of histone hypercitrullination, PAD4 mediates chromatin decondensation, and the DNA-protein complexes are released extracellularly as NETs (Fuchs et al., 2007). Thus, whereas plasma membrane integrity remains, both the nuclear membrane and granular membrane degenerate during NETosis (Yousefi et al., 2019). If successful, in the end, the organisms are trapped in these NETs and killed by the coordinated action of enzimes such as MPO, NE and cathepsin G and ROS products. On the other hand, excessive formation of NETs or ineffective clearance can induce pathological effects, such as endothelial dysfunction, pro-inflammatory effects, thrombosis by stimulating platelet aggregation, and thrombin generation (Papayannopoulos et al., 2010).

In many tissues, chronic inflammation induced by the presence of T-cells or macrophages is preceded by neutrophil infiltration. Neutrophil infiltration is a transient and premature stage in the inflammatory process, preparing for the recruitment and activation of other cell types. Blood neutrophils are vital in innate immunity since they constitute the most significant proportion of white blood cells (Jaillon et al., 2013).

Beyond their rapidly and classical secreted mediators, neutrophils have lately emerged as critical regulators in innate and adaptive immunity through cytokine production and secretion. Neutrophils promote the liberation of C-C motif chemokine ligand 2 (CCL2) and other cytokines, such as tumor necrosis factor alpha (TNF-α) (Jaillon et al., 2013). In adipose tissue, inflammatory markers secreted by macrophages further mobilize neutrophil migration into fatty tissue. These neutrophils, in turn, secrete cytokines that recruit more myeloid, T- and B-cells (Mraz and Haluzik, 2014). Adipokines are central to obesity, insulin resistance (IR), immunity, and inflammation. Of the adipokines, leptin has pro-inflammatory effects, whereas adiponectin has anti-inflammatory properties. (Lustig et al., 2022).

Cytokines play a relevant role during viral infections; thus, the host-viral relationship occurs through the activation of toll-like receptors (TLRs) and the identification of pathogen associated molecular patterns (PAMPs) (Kolli et al., 2013). The term “cytokine storm” was first coined in 1993 to describe a graft-versus-host disease. The term has since been extended to describe the sudden release of similar cytokines associated with autoimmune, hemophagocytic lymphohistiocytosis, sepsis, cancer, acute immunotherapeutic responses, and infectious diseases (Ferrara et al., 1993; Shimabukuro-Vornhagen et al., 2018).

Elevated inflammatory markers and increased serum levels of cytokines and chemokines favor the development of the severe form of COVID-19. Patients with the severe form of the disease had higher inflammatory biomarkers, such as C-reactive protein, lactic dehydrogenase, serum ferritin, interleukin (IL)-6, IL-1β, IL-1Rα, IL-7, IL-8, IL-10, basic fibroblast growth factor, granulocyte colony-stimulating factor, granulocyte-macrophage -CSF, interferon (IFN-gamma), induced protein (IP) -10 / CXCL10, MCP-1 / CC motif chemokine ligand (CCL) -2, macrophage inflammatory protein (MIP)-1α / CCL3, MIP-1β/CCL4, platelet-derived growth factor, TNF-α and vascular endothelial growth, suggesting that a cytokine storm underpins severe COVID-19 immunopathology (Gustine and Jones, 2021).

In COVID-19, the accumulation of neutrophils generates a toxic environment that contributes to the pathophysiology of severe acute respiratory syndrome. In this context, neutrophils are activated and induce the release of ROS, superoxide, and hydrogen peroxide (H_2_O_2_), causing oxidative stress, which contributes to the cytokine storm and blood clot formation in SARS-CoV-2 infection (Borges et al., 2020; Saleh et al., 2021).

Among the processes triggered by neutrophils in COVID-19, NETs are released having neutrophil elastase (NE) as one of the leading members of their networks, a proteolytic enzyme stored in azurophilic granules, secreted to degrade proteins (Middleton et al., 2020). An imbalance of NE and other proteinases induces damage to the alveolar-capillary barrier, resulting in tissue damage and edema formation (Jorch and Kubes, 2017).

The increase in plasma NETs is associated with increased severity of COVID-19, as well as lung injury and microvascular thrombosis (Tomar et al., 2020). This harmful effect of NETs is also reported in other organ tissues, such as the kidneys and the liver and it may be associated with thrombus triggering and reinforcing neutrophil association in the immunopathology of COVID-19 (Veras et al., 2020).

Glucose is the primary substrate used by neutrophils in physiologic conditions. Neutrophils take glucose *via* glucose transporter-1 (GLUT-1) (Maratou et al., 2007), which is subsequently converted to glucose-6-phosphate by the enzyme hexokinase. This molecule is then converted through a series of reactions to pyruvate *via* the glycolytic pathway. In this process, there is generation of adenosine triphosphate (ATP), and the reduced form of nicotinamide adenine dinucleotide (NAD), namely NADH. In neutrophils, pyruvate is directed to lactate synthesis, following the anaerobic glycolytic pathway (Curi et al., 2020). Hypoxia-induced transcription factor (HIF)-1α is one of the main transcriptional regulators of genes in neutrophils during hypoxia. It is essential for allowing cell adaptation in hypoxic conditions. This transcription factor induces cell survival *via* NF-kB-dependent HIF-1α, which is necessary for inflammation resolution (McGettrick and O’Neill, 2020). Codo et al. showed that the mitochondrial ROS/HIF-1a/glycolysis axis is induced in monocytes and macrophages infected with SARS-CoV-2, which allows a higher production of pro-inflammatory cytokines and interferons (IFNs) (Codo et al., 2020). Additionally, glucose enhances both viral replication and cytokine synthesis. Therefore, it is hypothesized that this axis may be increased in neutrophils during infection by SARS-CoV-2.

Glycolysis is the main pathway to ATP production for neutrophil functions, such as phagocytosis and NET formation (Curi et al., 2020), which were reported to have increased rates in COVID-19 patients (Veras et al., 2020), and enriches the idea that glucose may be highly required in SARS-CoV-2 infection. In contrast to glycolysis, the pentose phosphate pathway is an alternative route for glucose-6-phosphate, in which this molecule is converted to ribose-5-phosphate and nicotinamide adenine dinucleotide phosphate (NADPH) in neutrophil cytosol and proceeds to DNA and RNA production (Kumar and Dikshit, 2019).

NADPH production in neuthophil is indispensable for the cytosolic NADPH oxidase (NOX) ROS production (Injarabian et al., 2019). Violi et al. reported an overreaction of NOX2 in hospitalized COVID-19 patients. They observed a high NOX2 plasma concentration in Intensive Care Unit (ICU) COVID-19 patients and even higher in thrombosis cases, which implies NOX2 rates relate to a poor diagnosis (Violi et al., 2020).

Glutamine is an amino acid with relatively high concentration in the blood. Castell et al. described glutamine in human neutrophils (Castell et al., 2004) and we were the first to report glutamine presence in neutrophils at high concentrations and its contribution to neutrophil functions. Curi-Pithon et al. observed high concentration of intracellular glutamine in Wistar rats’ neutrophils. Also, using *in vitro* assays we reported that the glutamine metabolism preserved the function of neutrophils (Pithon-Curi et al., 1997, 2002b). Different pathways in the neutrophil are associated with glutamine: DNA and RNA production, cytokines production, ROS, and apoptosis (Cruzat et al., 2018). In neutrophils from COVID-19 patients, these pathways are dysregulated. Therefore, glutamine must be an important factor for those neutrophil functions reestablishment (Kumar and Dikshit, 2019; Curi et al., 2020). Additionally, in pathologic situations, when neutrophils are deprived of glucose, they can change their energy substrate to glutamine (Injarabian et al., 2019).

Glutamine metabolism involves two enzymes, glutaminase phosphate dependent (GLS) and glutamine synthetase (GS). GLS is responsible for glutamine hydrolysis, converting it to glutamate-ammonia NH_4_. At the same time, GS activates the ion production glutamine ammonia NH_4_ (Pithon-Curi et al., 2002a; Hatanaka et al., 2006; Tan et al., 2017). In clinical settings in the COVID-19 risk group, a history of decreased glutamine levels and increased hexosamine levels has been seen (Matsuyama et al., 2021).

### Lymphocytes

Lymphocytes can be divided by their function into two main populations: B and T lymphocytes. B lymphocytes participate in the humoral response and, if activated, are differentiated into plasma cells, which secrete antibodies (Luan et al., 2014). T lymphocytes are involved in cellular immunity, modulate the immune response in the presence of chemical mediators, and participate in the activation of other immune system cells (Rubel et al., 2010).

T lymphocytes are fully activated when a foreign peptide is recognized or in the presence of some inflammatory conditions. In this pro-inflammatory environment, costimulatory ligands and increased expression of MHC class I and II molecules are induced in antigen-presenting cells (APCs), which are necessary for activation of T lymphocytes and cytokines that attract T lymphocytes, activating them through their antigenic receptors (Steinman et al., 2012).

When stimulated, lymphocytes can differentiate into different profiles, proinflammatories, such as Th1 (Th1) and Th17 lymphocytes (Th17), cells related to hyper sensibility, such as Th2 (Th2) lymphocytes and the immunosuppressor regulatory T lymphocytes (Treg). Th1 cells are involved in eliminating intracellular pathogens and are associated with organ-specific autoimmunity (Prete, 1992). They mainly secrete IL-2, IFN-gamma, TNFα, and IL-6. IFN-gamma is essential for activating mononuclear phagocytes, including macrophages and microglial cells, thus resulting in increased phagocytic activity (Murray et al., 1985).

Th2 lymphocytes are known for their association with allergic reactions (Sokol et al., 2009). They mainly secrete the cytokines IL-4, IL-5, and IL-10, which have anti-inflammatory action. Of these, the cytokine most produced by these cells is IL-4, which favors the differentiation of Th0 cells into Th2 cells. Th1 and IL-4 also upregulate the low-affinity IgE receptor in B lymphocytes and mononuclear phagocytes and the high-affinity IgE receptor in mast cells and basophils (Prete, 1992; Steinke and Borish, 2001; van Panhuys et al., 2008).

Th17 lymphocytes induce inflammation and autoimmunity and mainly produce the cytokines IL-22, IL-21, and IL-17. IL-17 leads to the induction of proinflammatory cytokines, including IL-6, IL-1β, TNF-α, and proinflammatory chemokines, ensuring the chemotaxis of neutrophils and other immune cells to sites of inflammation (Moseley et al., 2003; Ivanov et al., 2006).

Treg cells can be divided into a natural subset derived from the thymus with FOXP3 expression and Treg cells induced in peripheral tissues, which arise from naive CD4+ CD25+ cells after antigen stimulation in a suitable cytokine medium (Chen et al., 2003). Its central effector cytokines include IL-10, TGF-beta, and IL-35. IL-35 plays a vital role in the immune system as a cytokine inhibitor. It can modulate the T cell functions, activate bone marrow-derived immunosuppressive cells and regulate the actions of an inflammatory factor related to the immune system. Therefore, the regulation of IL-35 is of great importance in chronic diseases (Zhang et al., 2019).

The metabolic changes in leukocytes are associated with essential changes in their phenotypes and functions. The naïve lymphocytes in the lymphoid tissues and the bloodstream are metabolically less active. Upon contact with pathogens or neoplastic cells, these cells are activated, proliferating and secreting cytokines to coordinate the immune response. The activation of these cells is accompanied by metabolic changes in biosynthetic and energetic pathways which are stimulated (Buck et al., 2015).

An increased metabolic rate is essential to ensure that all processes involved in the immune response function correctly and effectively. The metabolism of T cells changes during the process of activation, proliferation, and differentiation. T cells need to reprogram their metabolic pattern to meet their bioenergetic and biosynthetic needs, using different metabolic substrates (glucose, amino acids, and fatty acids) and activating a given metabolic pathway in each situation (glycolysis, oxidative phosphorylation, pentose phosphate pathway, synthesis and fatty acid oxidation and glutamine metabolism) (Gerriets and Rathmell, 2012; Jung et al., 2019). Through these mechanisms, lymphocytes can provide an appropriate scenario for the synthesis of macromolecules and organelles to perform the cell division process. The metabolic pathways responsible for synthesizing DNA, RNA, and structural lipids such as phospholipids and cholesterol must have high activity and an adequate energy supply in the form of ATP (Curi et al., 1993).

Highly proliferation cells, such as activated lymphocytes, use high levels of glucose and glutamine, but the oxidation of these metabolites is low. Glucose is mainly converted into lactate and glutamine into glutamate, aspartate, and lactate (Ardawi and Newsholme, 1983).

T lymphocytes in a quiescent state produce ATP through beta-oxidation of fatty acids (FAs) and use pyruvate derived from glucose *via* oxidative phosphorylation (OXPHOS) (Buck et al., 2015; Rangel Rivera et al., 2021). When antigens are presented together with the MHC molecules and co-stimulatory signals, these cells are activated and proliferate, increasing their energy demand.

During the T lymphocyte activation process, to meet the increased metabolic demand, there is an increase in the expression of GLUT1 (Frauwirth et al., 2002; Buck et al., 2015). After the activation of T lymphocytes, several transcription factors and signaling pathways regulate metabolic reprogramming, such as IL-2, the activation of mammalian target of rapamycin (mTOR) complexes, and the Akt pathway. The cells then mainly use glycolysis and amino acids such as glutamine to generate ATP (Chang et al., 2013; Buck et al., 2015; Rangel Rivera et al., 2021).

From the activation of T cells, there is the mechanism of differentiation of these cells from stimuli of different cytokines and metabolic pathways (Michalek et al., 2011; Buck et al., 2015). So, cells with Th1, Th2, and Th17 features tend to use the glycolytic pathway more through mTOR signaling, while Treg cells preferentially use the FAs oxidation pathway. Signaling through mTORC1 and mTORC2 favors the differentiation of cells with Th1 and Th2 profiles (Delgoffe et al., 2011). Short-chain FAs and retinoic acid can induce the differentiation of Tregs in synergy with TGF-β (Coombes et al., 2007; Schmidt et al., 2016). The vitamin shortage, in turn, may inhibit the cellular immunity of Th1 and Th17 lymphocytes (Hall et al., 2011; Buck et al., 2015) in immune cells and promote exacerbated inflammatory responses.

Several studies over the years have shown increased markers associated with immunosenescence in obese individuals, generating a state of premature aging of immune cells (Shirakawa et al., 2016; Brunelli et al., 2022). The development of obesity can generate shortening of telomeres due to increased replicative demands. The increased metabolic load that occurs in obesity favors mitochondrial dysfunction, attenuating the functions of the electron transport chain (ETC) and generating an increase in the production of ROS (Hey-Mogensen et al., 2012; Shirakawa et al., 2016; Schafer et al., 2017).

### Macrophages

Macrophages are highly plastic cells capable of rapidly changing their functional profile through a process defined as polarization in response to the stimulus in the local microenvironment. Usually, these cells are classified as classically activated (pro-inflammatory or M1 macrophages) (Nathan et al., 1983; Pace et al., 1983) or as alternatively activated (anti-inflammatory or M2 macrophages) (Stein et al., 1992; Doyle et al., 1994). Each subtype presents a specific gene expression program, leading to the acquisition of different markers on the cell surface, secretion of cytokines, as well specific metabolic adaptations.

Pro-inflammatory macrophages are activated by microbial products, such as lipopolysaccharide (LPS) and other ligands of TLRs, and produce pro-inflammatory cytokines in large amounts, such as TNF-a, IL1-β, IL-6, IL-12, and IL-23 (Mosser and Edwards, 2008). Glycolysis (Freemerman et al., 2014) and the pentose-phosphate pathway (Tannahill et al., 2013) are the primary sources of ATP for pro-inflammatory macrophages, while the Krebs cycle is broken at two points (Newsholme et al., 1996; Peres et al., 1999; Meiser et al., 2016). Oxidation of fatty acids (FAO) is downregulated (Feingold et al., 2012).

M2 or anti-inflammatory macrophages are induced by IL-4 or IL-13 secreted by adaptive and innate immune cells such as Th2 lymphocytes, basophils, and mast cells (Stein et al., 1992). In M2 macrophages, the metabolic requirement is mainly supported by the Krebs cycle with increased FA oxidation and OXPHOS activity (Jha et al., 2015).

Macrophage Activation Syndrome was described as a serious risk factor contributing to lung inflammation, acute respiratory distress syndrome (ARDS), and subsequent death of COVID-19 patients (Giamarellos-Bourboulis et al., 2020; Toor et al., 2020). Autopsies in patients who died of COVID-19 revealed a high infiltration of macrophages within the area of bronchopneumonia (Barton et al., 2020). SARS-CoV-2 enters the host cells *via* ACE-2 receptor interaction, which is present in several cells, including macrophages.

The Warburg effect likely supports SARS-Cov-2 replication in cells expressing ACE-2 (Icard et al., 2021). This hypothesis is reinforced by studies showing that the increased flux through glycolysis supports virus replication in colon cancer cells and blood monocytes (Bojkova et al., 2020; Codo et al., 2020). In monocytes, SARS-CoV2 replication and the induced cell response are sustained by the switch to aerobic glycolysis (the Warburg effect) (Codo et al., 2020). In addition, these authors observed that the infection increase mitochondrial ROS production, leading to (HIF-1α) stabilization that may favor glycolysis major recruitment.

## Metabolic changes in obesity, type 2 diabetes, and the immune system

Obesity was soon identified as a risk factor for the worse prognosis of COVID-19 (Wu et al., 2020), including the occurrence of ARDS, in addition to adverse cardiovascular events in up to 28% of patients hospitalized (Guo et al., 2020). The prevalence of obesity is increasing worldwide and is currently considered a significant public health problem because it affects billions of people. The role of ectopic fat deposits has attracted interest in the COVID-19 scenario because this increase in adiposity may be related with a poor prognosis of disease in patients (Hoffmann et al., 2020).

During weight gain, adipose tissue undergoes multiple processes of structural and cellular remodeling. First, mature adipocytes expand during the chronic positive energy balance, becoming hypertrophic to store more fat. If this extra energy is not used, the number of cells increases (hyperplasia) (Haczeyni et al., 2018). Hyperplastic and hypertrophic adipocytes are usually hypoxic, partially explaining the development of inflammation (Castoldi et al., 2016). Subsequently, hypoxia induces the activation of HIF-1α, which acts as a critical regulator of physiological functions, including metabolism, cell proliferation, and angiogenesis (Allen et al., 2020; Ménégaut et al., 2020). HIF-1α activates glycolysis and inflammatory response, which implies the effects of HIF-1α on the pathogenesis of COVID-19. HIF-1α leads to a potent profibrotic transcription program with extracellular matrix components (ECM) accumulation, leading to fibrosis and adipose tissue dysfunction (Lippi and Henry, 2020; Zhou et al., 2020b). Tian et al. recently demonstrated that during SARS-CoV-2 disease, the viral protein ORF3a increases the production of HIF-1α, which promotes SARS-CoV-2 disease and inflammatory responses (Tian et al., 2021).Simultaneously, immune cells infiltrate adipose tissue, and proinflammatory cytokines are overexpressed (Xydakis et al., 2020). A thin individual has a high proportion of M2/M1 macrophages, eosinophils, and regulatory T cells, which secrete ILs −4/−13 and − 10, leading to an anti-inflammatory phenotype. This scenario is different in the obesity condition, in which there is a metabolic disorder that involves excessive fat accumulation by the adipose tissue and various tissues, organs, and systems, causing hormonal imbalance in cells, metabolic pathways, vessels, and arteries (Xydakis et al., 2020). This low-grade chronic inflammatory and metabolic disease can alter the mechanisms of innate and adaptive immune responses, increasing susceptibility to infections and other diseased conditions, such as DM2, cardiovascular diseases, hypertension, and some types of cancer (Xydakis et al., 2020).

In obesity, there is an activation of several stress pathways, such as endoplasmic reticulum stress, oxidative stress, and inflammasome complexes (Hotamisligil, 2010), in addition to tissue hypoxia, which induces a change in innate immunity and lymphoid cells and a modification of the macrophage signature with a rapid shift in polarization toward an M1 phenotype, associated with adipose tissue inflammation and insulin resistance (IR) (Lumeng et al., 2007; Dalmas et al., 2011; Castoldi et al., 2016). A low-grade state of chronic inflammation is, therefore, mainly explained by the imbalance of immune cells in a dysfunctional adipose tissue. Stressed adipocytes release fatty acids (FAs) and secrete chemokines that lead to the infiltration of inflammatory immune cells that secrete proinflammatory cytokines (Xu et al., 2015). In addition, dysbiosis of the intestinal microbiota can also trigger inflammation by activating immune signaling pathways (Cox et al., 2015).

In patients with obesity, in which the white adipose tissue (WAT) is increased, and brown adipose tissue (BAT) is decreased (Zhou et al., 2020b), the renin-angiotensin-aldosterone system (RAAS) is chronically activated. This alteration predisposes to several dysfunctions, including cardiac pathologies and renal dysfunction. These changes are associated not only with hypertension (Hoffmann et al., 2020) but also with insulin signaling in peripheral tissues (Donoghue et al., 2000), the inflammatory state of the pancreas, and the β cell death profile (Yuan et al., 2010). The increase in oxidative stress is possibly the basis of the cytotoxic effects induced by angiotensin II and aldosterone during the exaggerated activation of SARS-CoV-2 (Luther et al., 2011). The resulting IR acts as an impetus for the progression of cardiometabolic syndrome, which is commonly associated with obesity (Jing et al., 2013). There is then induction of the ACE2 protein axis. ACE2 is a type I transmembrane glycoprotein of 805 amino acids (~ 120 kDa) containing a single extracellular catalytic domain whose sequence is 41.8% identical to the ACE domain (Hamming et al., 2007). It is associated with the activation of BAT and the darkening of WAT, which are related to anti-obesity effects (Kawabe et al., 2019). Due to many changes in physiology during obesity, including RAAS dysfunction, BAT tends to decrease in size and activity, increasing the chance of comorbidities (Shimizu and Walsh, 2015). RAAS components, including ACE2, are expressed in adipocytes and are crucial for the homeostasis of glucose and lipid metabolism. The entry of SARS-CoV-2 into the host cell depends on the ACE2 receptor. Under obesity, adipocytes express more ACE2, allowing the adherence of the virus to the cells (Jing et al., 2013). Experiments in mice showed that obesity induced by a high-fat diet is associated with increased expression of ACE2 in adipose tissue (Patel et al., 2016). In an elegant study, Pasquarelli et al. indicated that the increased expression of ACE2 in adipose tissue of obese individuals may have consequences for SARS-CoV-2 infection (Pasquarelli-do-Nascimento et al., 2020). In addition, obesity causes hyperglycemia through IR, while there is increasing evidence that SARS-CoV-2 can also cause hyperglycemia by infecting and killing β cells (Yang et al., 2020).

DMs occur after a period characterized by reduced insulin sensitivity, also known as IR, chronic hyperglycemia, and consequent pancreatic beta cell dysfunction with possible progression to cell death and disease onset (Kahn, 2003; Reed et al., 2021). DM is one of the main noncommunicable diseases in the world and is considered a public health problem (Herman et al., 2012). DM can be classified by its etiology, as proposed by the WHO and the American Diabetes Association (ADA) and recommended by the Brazilian Diabetes Society, being named, for example, DM type (DM1) and type 2 DM (DM2), the two most common types of DM observed in the population (Boura-Halfon and Zick, 2009).

Chronically, DM can lead to chronic complications, especially micro- and macro-vascular complications, such as retinopathy, neuropathy and vasculopathy, urological diseases, and cancer (Vikram et al., 2014).

Several studies have shown that DM constitutes the main comorbidity in patients with severe disease admitted to the ICU for COVID-19 compared to those patients with mild symptoms (Deng and Peng, 2020; Wang et al., 2020; Zhou et al., 2020a).

Patients with preexisting DM2 also presented worse prognosis in SARS-CoV infection (Booth, 2003; Yang et al., 2006; Gorjão et al., 2022). Epidemiological studies also indicated that DM2 is the primary comorbidity associated with severe or lethal MERS-CoV infections (Al-Qahtani et al., 2018).

In DM2, the inflammatory response occurs due to the immune response to high blood glucose levels and the presence of inflammatory mediators produced by adipocytes and macrophages in adipose tissue. This low-grade chronic inflammation damages pancreatic beta cells and causes insufficient insulin production, which results in chronic hyperglycemia. Hyperglycemia in DMs can cause immune response dysfunction, which fails to control the spread of invasive pathogens in diabetic individuals, making them more susceptible to infections. Thus, there is a risk that infectious diseases are more severe in diabetic patients. In addition, as already mentioned, diabetic patients tend to have long-term changes, especially in organs such as the kidneys and heart. This association with hypertension and obesity also exacerbates infections, such as COVID-19 (Berbudi et al., 2020; Connors and Levy, 2020; Wilk et al., 2020).

Zeng et al. (2012) identified higher proportions of proinflammatory CD4+ T cells and circulating memory in DM2 diabetic patients than in nondiabetic patients. The proportion of Treg cells was lower and the ratio of Treg to Th1 and Th17 cells was decreased in diabetic patients compared to nondiabetic patients, suggesting a shift toward a proinflammatory CD4+ T-cell profile.

Hotamisligil (2010) observed an increase in TNF-α in the adipose tissue of different animal models (db/db) with obesity and diabetes. Neutralization of TNF-α lead to an improvement in peripheral glucose uptake indicating a role of this cytokine in the phenotype. In addition to TNF-α, other cytokines such as IL-1β and IFN-gamma are increased in obesity and DM2 and contribute to the impairment of the insulin signaling response (Ouchi et al., 2011; Mathis, 2013). In contrast, anti-inflammatory cytokines such as IL-4 and IL-10 are associated with protection against insulin sensitivity (Odegaard et al., 2007).

The hyperglycemia associated with the imbalance of the immune response in DM2 results in the inability to resolve the infection in diabetic individuals, generating chronic stimulation of the M1 profile macrophages. Macrophage functions and glucose metabolism are closely connected. An increase in glucose metabolism by macrophages, such as in DM, may influence metabolic reprogramming and the immune response capacity. In addition, as other authors reported, some kinases are involved in glucose metabolism and the immunomodulatory role of macrophages (Mantovani, 2008; Mauer et al., 2014; Brady et al., 2016).

## Covid-19, immune function, diabetes, and obesity

The infection itself is not limited to the lung tissue since after the entry of the virus through the airways, alveolar macrophages become secondary targets, which can initiate the cytokine storm, thus causing severe acute respiratory syndrome with subsequent respiratory failure (Almerie and Kerrigan, 2020). Due to the increased viral load in the upper respiratory tract cells, these cells dye thus releasing viral particles capable of infecting other cells and tissues.

As an example, they infect the cells of the lower respiratory tract, intestinal tract, heart, blood vessels, kidneys, and urinary bladder, which express high amounts of ACE2 and TMPRSS2. Therefore, there is a considerable worsening of the disease due to the death of infected cells, tissues and organs, mainly due to the exacerbated increase in secreted proinflammatory cytokines (IL-6, IL-1β, TNF-α, IFN-gamma, G-CSF, and CCL3) (Almerie and Kerrigan, 2020).

Obese individuals have WAT in the large walls of the airways in proportion to body mass index (BMI), which can lead to airway thickening, infiltration of immune cells, tissue damage, and pulmonary fibrosis (PF) (Kruger et al., 2015; Elliot et al., 2019). The increased expression of ACE2 in WAT during obesity makes these intrapulmonary deposits susceptible to SARS-CoV-2 infection in the lung tissue. In addition, the prolonged viral dissemination would facilitate lung damage and consequent respiratory failure in cases of obesity (López-Reyes et al., 2020).

Adipose cells called lipofibroblasts (LiFs) affect lung function because the transdifferentiation of these cells into myofibroblasts, which leads to PF (Siripanthong et al., 2020). The LiFs have lipid droplets in their cytoplasm containing high levels of perilipin-2. Located in the alveolar interstitium, these cells reside in the vicinity of type 2 alveolar epithelial cells (AEC2) that express ACE2, to which they provide surfactant molecules. AEC2 is considered the largest pool of cells that express ACE2 in the lungs, and the proximity of LiFs may indicate a greater chance of PF in the lungs of infected obese individuals (Stefanini et al., 2020). In addition, the possibility of LiFs expressing ACE2 should be considered since PF is a common characteristic among deceased patients with COVID-19 (Stefanini et al., 2020).

COVID-19, therefore, induces an immune-mediated inflammatory response. In this context, epicardial adipose tissue can transduce this inflammation to the heart. Thus, the inflammation of this tissue may be implicated in COVID-19 myocarditis due to its contiguity with the myocardium and its proinflammatory secretome reaching the myopericardium directly through the blood vessels and paracrinally (Lasbleiz et al., 2021).

Some studies reported that the expression of ACE2 in adipocytes is higher than in lung cells, making these cells an important viral reservoir (Gupte et al., 2008; Haczeyni et al., 2018). High-fat fet mice show increased expression of ACE2 in adipocytes (Maier et al., 2018). In obesity, excess adipose tissue may increase SARS-CoV-2 infection and tissue accessibility, leading to increased systemic viral spread and prolonged viral entry and spread (Qin et al., 2020), as seen during the influenza A epidemic.

An interesting point to be explored is the infection of adipose tissue with SARS-CoV-2, which can potentially increase proinflammatory cytokine secretion by this tissue (Martínez-Colón et al., 2022; Saccon et al., 2022). In 2022, Basolo et al. (2022) observed that the nucleocapsid antigen SARS-CoV-2 was significantly detected in adipocytes from subcutaneous abdominal adipose tissue samples of patients who died of severe COVID-19, suggesting that the virus can directly infect subcutaneous fat depots. The anatomical localization of adipose tissue also can directly impact the susceptibility of SARS-CoV-2 infection, as demonstrated by Saccon et al. (2022). These authors observed high expression of ACE2 in visceral fat depots, suggesting elevated susceptibility to the SARS-CoV-2 infection. In addition, visceral fat cells present higher expression of pro-inflammatory cytokines than subcutaneous fat cells. It was demonstrated that when these fat cells are infected with the gamma variant, there is upregulation of proteins involved in the IFN signaling pathway, leading to a much softer induction of pro-inflammatory markers in comparison to the ancient SARS-CoV-2 lineage.

It has been described that the replication of SARS-CoV-2 in adipose tissue cells and its inflammatory insult are favored by the presence of lipid droplets. The hypothesis is that these lipid droplets function as reservoirs for viral replication and favor the production of proinflammatory cytokines (Dias et al., 2020; Ryan and Caplice, 2020; Saccon et al., 2022). In addition, adipokines are also influenced by COVID-19. Tonon et al. (2022) observed that patients with COVID-19 in severe condition present high levels of leptin and low adiponectin/leptin ratio, associated with increased expression of IL-6. Reiterer et al. (2021) demonstrated that SARS-CoV-2 in the adipose tissue of hamsters have decreased adiponectin protein level, but no mRNA levels alteration, suggesting the involvement of a post-transcriptional mechanism in this process. Thus, the characteristics of adipose tissue cells are determinant for the SARS-CoV-2 susceptibility and effects, including anatomical localization, ACE2 expression, pro- and anti-inflammatory cytokine profile, lipid droplet amount, and adipokine production, which can lately and negatively impact the metabolic responses in type 2 diabetic and obese patients.

After infection of host cells, the recruitment of proinflammatory cytokines and impaired T lymphocytes culminates in a cytokine storm associated with progression to ARDS and multiple organ failure (Dalmas et al., 2011). In severe respiratory forms, patients with COVID-19 infection exhibited macrophage activation syndrome. There is a decrease in CD4+ and CD8+ T lymphocytes (Giamarellos-Bourboulis et al., 2020) but a higher proportion of pro-inflammatory Th17 cells and of pro-inflammatory cytokines such as IL-2, IL-6, and TNF-α (Mirsoian et al., 2014; Chen et al., 2020).

Lymphopenia, is related to a 3-fold higher risk of severe COVID-19 infection (Zhao et al., 2020), and several mechanisms may be involved in SARS-CoV-2-induced depletion and exhaustion of lymphocytes. First of all, SARS-CoV-2 may infect T cells *via* ACE2 receptor expressed on T cells (Kuklina, 2022), which promotes T cell death (Yue et al., 2018). Secondly, several cytokines (anti-inflammatory or pro-inflammatory) can accelerate the exhaustion and depletion of T cells, impacting on their respective roles. Besides, the virus may destroy lymph nodes and secondary lymphoid tissues such as spleen, leading to lymphopenia, which is reinforced by the observations of lymph node necrosis, splenic atrophy, and reduced lymphocyte numbers (Li et al., 2020; Tan et al., 2020). As previously mentioned, in obesity, dysfunctional hypertrophic adipocytes produce more pro-inflammatory cytokines, leading to a low-grade state of chronic inflammation. This state, in turn, causes metabolic and immunological disorders, making a cytokine storm more likely (Wang et al., 2019).

On the other hand, COVID-19 patients who were admitted to the ICU had a greatly elevation in blood neutrophil counts as compared to other SARSCoV2-positive patients with less severe symptoms (Huang et al., 2020). Cohort studies with COVID-19 patients also described neutrophilia and sustained low levels of lymphocyte counts, conducting to a high neutrophil-to-lymphocyte ratio (NLR), which is predictive of severe illness in the early stage of SARS-CoV-2 infection (Zhu et al., 2020).

A significant increase in the plasma level of IL-1β was reported in COVID-19 patients (Darif et al., 2021), suggesting that the NOD-, LRR- and pyrin domain-containing protein 3 (NLRP3) may be involved in the pathogenesis of pulmonary infection and injury. NLRP3 is a multiprotein complex in macrophages, dendritic cells, and other non-immune cells. The activation of NLRP3, a central component of the innate immune system, plays a key role in host defense but is also associated with metabolic and inflammatory conditions (López-Reyes et al., 2020). During SARS-CoV-2 infection, intense and rapid stimulation of the immune system response can trigger the activation of the NLRP3 inflammasome pathway and the release of its products, including IL-18 and IL-1β (Freeman and Swartz, 2020; Amin et al., 2022), which may be involved in the maintenance of inflammation. Viral infection could potentiate this underlying systemic inflammatory state, partially explaining the worse progression of the disease in obese patients (Barra et al., 2020). There is a higher expression of ACE2 and TMPRSS2 in the pulmonary epithelial cells of individuals with obesity than in those without the disease, as demonstrated *in vitro* (Al Heialy et al., 2020). These conditions may contribute for the high occurrence of ARDS in obese individuals.

The platelets of obese individuals exhibit a series of abnormalities that contribute to the hypercoagulability state observed in them (Barrachina et al., 2019). Thus, an inherent exacerbated state of inflammation and a tendency to develop hypercoagulation are probably contributing to the higher mortality rates observed on COVID-19-infected obese individuals. Prothrombotic factors are positively related to visceral fat. People with obesity have higher plasmatic concentrations of all prothrombotic factors (factor VII, fibrinogen, and von Willebrand factor) than nonobese individuals (De Pergola et al., 1997). Similarly, plasma concentrations of PAI-1, a physiological inhibitor of plasminogen activators (urokinase and tissue types) synthesized by adipose tissue, are highly elevated in the plasma of obese individuals (Skurk and Hauner, 2004; Raiko et al., 2012), predisposing these individuals to thrombotic complications. All these conditions contribute to the progression of the prothrombotic state reported in obesity.

In patients with DM2, there is an increased risk of severity and mortality associated with COVID-19 (Holman et al., 2020). It is established that patients with DM2 are more susceptible to infections in general and have a worse prognosis when infected (Kumar Nathella and Babu, 2017; Xu et al., 2020). These patients have increased risk for bacterial, mycotic, parasitic, and viral infections. High susceptibility in the diabetic population was also observed in other pandemics resulting from coronaviruses such as MERS and SARS-Cov (Booth, 2003; Yang et al., 2006).

The impaired function of T cells and high levels of IL-6 also play a relevant role in the progression of COVID-19 in diabetic patients (Kulcsar et al., 2019). T cells are essential in regulating antibody-mediated cellular immunity (humoral immunity). In 2021, Zheng et al. (2021) demonstrated immunological changes in patients diagnosed with DM and COVID-19. These patients showed an increased percentage of T CD4+ cells compared to the non-diabetic group, and a decreased number of T CD8+ cells. There was also an increase in cytokines such as IL-6, TNF-α, IFN-gamma, IL-2, and IL-10 compared to the non-diabetic group. The immune system imbalance results in chronic inflammation, which is a way for the body to respond to infections such as those caused by viruses.

Cytokine storms or excessive inflammatory reactions are serious complications in patients with SARS or MERS. Zhou et al. (2020a) identified increased IL-6 production in monocytes of patients with COVID-19. Studies suggest that the severity of COVID-19 is associated with elevated levels of inflammatory mediators; however, the elevation of IL-6 in the blood is highly correlated with the mortality caused by COVID-19 when survivors and non-survivors are compared (Liu et al., 2020; Zhou et al., 2020b). IL-6 is essential for the generation of Th17 cells. The increase in IL-6 may explain the rise in the Th17 profile found in patients with COVID-19, as reported by Xu et al. (2020).

**Figure 1:**
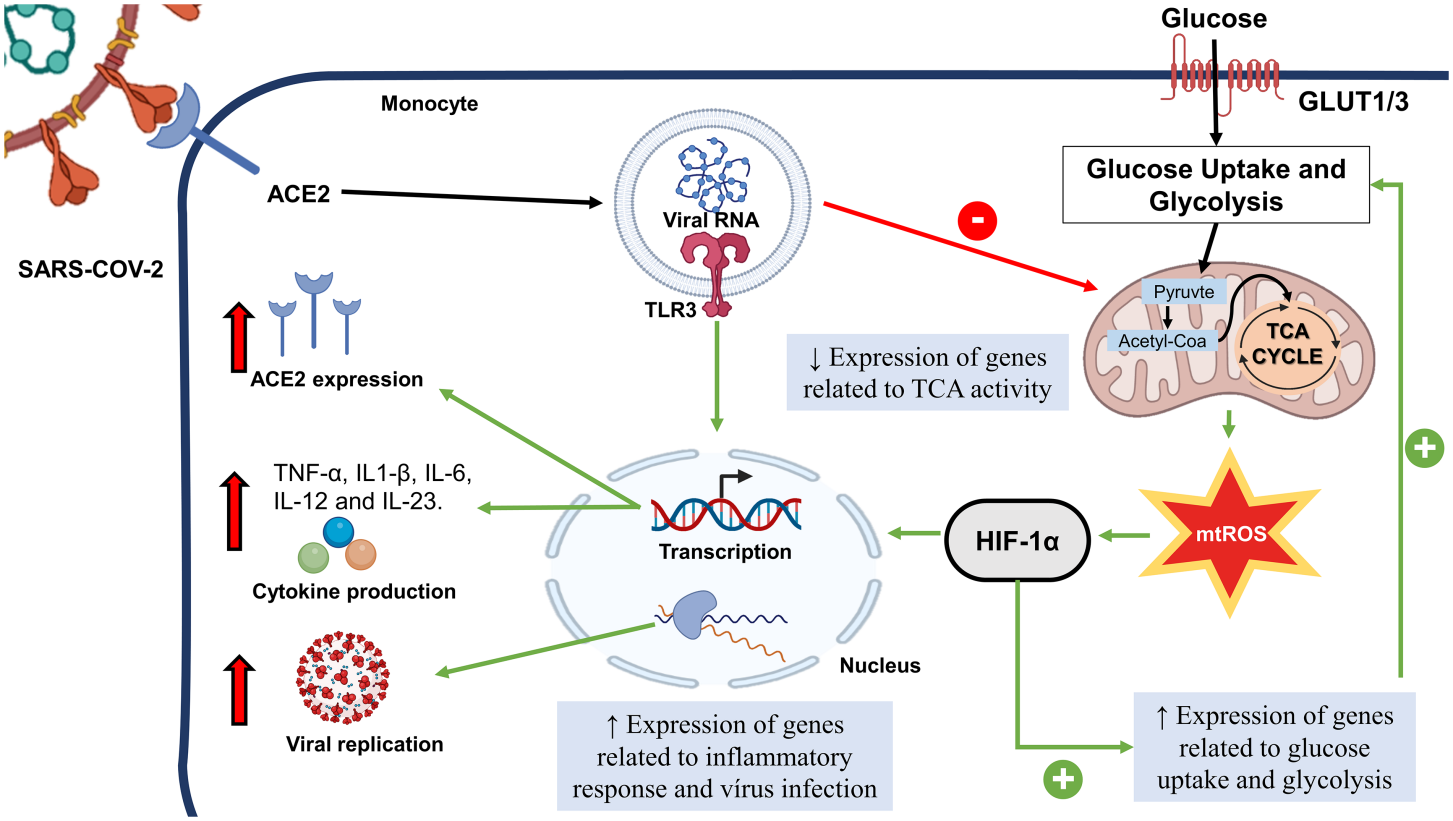
Possible Role of Sars-Cov-2 in Monocyte Metabolism. After the interaction of SARS-COV-2 with ACE2, the viral fragment binds to TRL3, leading to the decrease of genes related to TCA activity. There is a mtROS increase and HIF-1α activation, followed by the increase of genes related to glucose uptake. HIF-1α and TLR3 leads to transcription of genes related to pro-inflammatory cytokines, expression of ACE2 and genes related to response to virus infection and viral replication. SARS-CoV-2, Severe Acute Respiratory Coronavirus 2; HIF-1α, Hypoxia inducible factor-1 α; TCA, citric acid cycle; GLUT, glucose transporter; mTROS, mitochondrial reactive oxygen species; TLR, toll like receptor.

T-cell metabolism and function are closely related to metabolic reprogramming, which is crucial for T-cell activation. Notably, peripheral blood mononuclear cells (PBMCs) in an activated condition due to viral infection exhibit metabolic dysfunction characterized by increased glycolysis and reduced oxygen consumption (Ajaz et al., 2021). Monocytes infected with SARS-CoV-2 also show an increased glycolytic rate (Figure 1). Higher concentrations of glucose are associated with a boosting on SARS-CoV-2 replication in monocytes (Codo et al., 2020). In addition, the cytokines TNF-α, IL-1β, and IL-6, which were highly expressed in SARS-CoV-2 infected monocytes in elevated glucose levels, are related to T cell dysfunction and lymphopenia.

Because of this central role of glycolysis in the response of leukocytes in COVID-19, the glucose analog 2-deoxy-D-glucose (2DG), which inhibits glycolysis, underwent a phase III trial and received emergency treatment approval for severe COVID-19 in India. However, the study enrolled only 220 patients, and the data were unavailable to the public (Halder et al., 2021).

IFN-I and IFN-III are important for the intrinsic viral resistance of the cells. These antiviral mechanisms have been shown to be suppressed by coronavirus infection (Lim et al., 2016). In this sense, a study with COVID-19 patients showed reduced levels of type I and type III IFN as well as higher concentrations of cytokines and chemokines (Blanco-Melo et al., 2020). On the other hand, some studies suggested that the virus induced a late IFN action rather than a complete block of its production and effects on cells (Park and Iwasaki, 2020). Moreover, research with the SARS-CoV-infected mouse model showed that IFN-I was detectable in the lung for several hours after the viral load peak (Channappanavar et al., 2019). Also, in a study with a small cohort of COVID-19 patients was found a strong relationship between IFN-α and viral load and disease severity, concluding that high concentrations of IFN in the late phases of the infection were inefficient in decreasing the viral load and that IFN possibly was the most potent in the early phases of the disease (Wei et al., 2020).

Hyperglycemia can impair adaptive immunity through the induction of oxidative stress. Specifically, the increase in intracellular glucose concentration increases the mitochondrial proton gradient, releasing ROS through different sources (Rask-Madsen and King, 2013). Oxidative stress has a detrimental effect on CD8+ T-cell responses. Specifically, oxidative stress reduces the production of crucial effector cytokines, such as TNF-α and IFN-gamma, by peripheral blood T cells after stimulation with an MHC-I-specific influenza virus peptide (Malmberg et al., 2001). This effect is more pronounced in memory T cells (Hofstetter et al., 2016). Uncontrolled oxidative stress directs T-cell signaling and activation, potentially leading to its dysfunction.

Recently, the stress response at the cellular level was reorganized into a convergent signaling pathway called integrated stress response (ISR), which can be activated by multiple physiological and pathological situations or stressors, including hypoxia, viral infection, and intrinsic stress to cells, such as endoplasmic reticulum (ER) stress. The ISR signaling pathway is initiated when different stressors activate at least one member of a family of four serine/threonine kinases PKR-like ER kinase [PERK double-stranded RNA-dependent protein kinase (PKR)], heme-regulated eIF2a kinase (HRI), and general control nonderepressible 2 (GCN2) (Pakos-Zebrucka et al., 2016). A decrease in protein synthesis is caused by phosphorylation of E74-like factor 2 (elF2) and, at the same time, promotes cell survival and recovery, but the final response depends on whether cell stress is severe or not (Santos et al., 2021).

**Figure 2:**
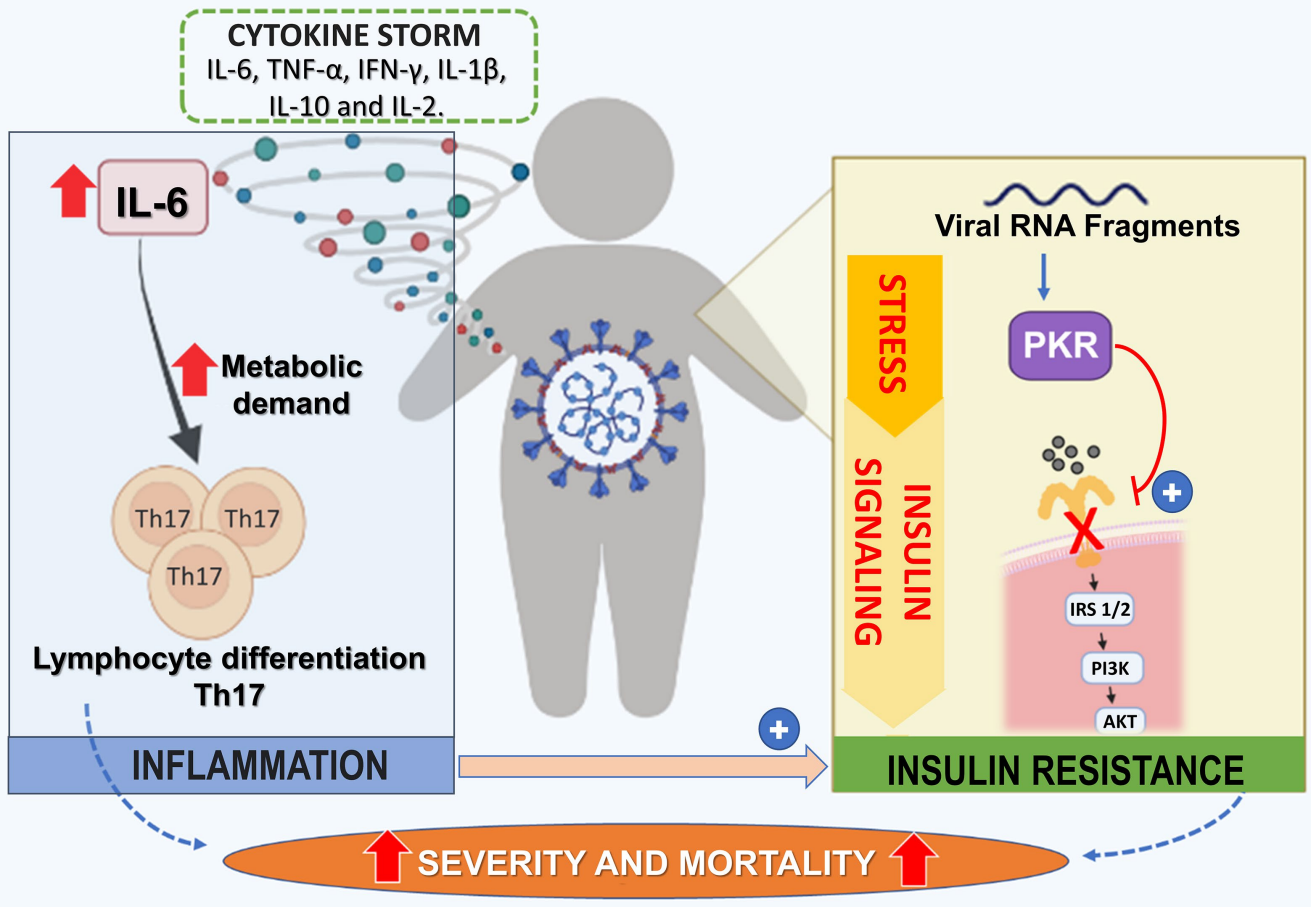
Potentiating Mechanisms of Virus Infection on Insulin Resistance State in Dm2 Patients. In patients with DM2 infected with COVID-19, there is an increase of cytokines, including IL-6, TNF-α, IFN-gamma, IL-1β, IL-10, and IL-2 leading to cytokine storm. IL-6 is related to the differentiation of Th-17 lymphocytes profile. This polarization process occurs with increased metabolic demand of lymphocytes, mainly through TCA and OXPHOS. On the other hand, the viral fragment of SARS-CoV-2 leads to activation of PKR (Protein kinase R), resulting in the serine phosphorylation of IRS-1/2, leading to the progression of insulin resistance. Together, cytokine storm and insulin resistance increase the risk of severity/mortality in individuals with DM2. TCA, citric acid cycle; OXPHOS, oxidative phosphorylation; PKR, Protein kinase R.

Specifically, for COVID-19, viral RNA fragments can activate PKR, which will induce serine phosphorylation of IRS-1 and IR (Figure 2). In addition, the cytokine storm and an increase in hormone signaling, such as cortisol, can activate some of the four kinases and contribute to IR. In addition, IR in adipose tissue can induce macrophage infiltration, leading to an inflammatory state. An important molecular mechanism associated with insulin signaling is the protein complex called mTORC2, which is activated by AKT and is an essential mediator of glucose metabolism and gene expression. An important gene suppressed by mTORC2 is the chemokine Ccl2, a key monocyte-chemoattractant (Huang et al., 2016). Thus, in IR, there is a reduction in mTORC2 and an increase in the infiltration of M1 macrophages into tissues. These data show that inflammation induced by IR may aggravate the cytokine storm characteristic of COVID-19 (Santos et al., 2021).

In CD4+ and CD8+ T cells, COVID-19 inhibits the activation of mTORC1, which reduces glycolytic activity, causing mitochondrial dysfunction and increased susceptibility to apoptosis (Liu et al., 2021). Accordingly, the expression levels of GLUT1 are decreased in the T cells of patients with severe COVID-19 compared to healthy controls or patients infected with influenza virus. However, there are contradictory results. De Biasi et al. showed that T cells of patients with COVID-19 have a similar capacity for metabolic reprogramming as compared to cells from uninfected patients (De Biasi et al., 2020).

In patients with DM (type 1 and type 2) (especially people with overweight and IR), COVID-19 can elevate IR. Even mild COVID-19 infection can promote pro-inflammatory responses, characterized by increased TNF-α, IL-10, IL-1β, IL-6, and Ccl2, leading to IR. Moreover, overweight, which is generally associated with DM2, elevates the cytokine response, exacerbating IR (Pal and Bhadada, 2020). SARS-CoV-2 also increases serum levels of fetuin-A, an a2 Hermans-Schmid glycoprotein associated with IR (Yamasandhi et al., 2021). Therefore, glycemia adjustment in hospitalized COVID-19 patients is vital, and the screening to identify undiagnosed cases of DM is markedly relevant (Hafidh et al., 2020). On the other hand, hypoglycemia promotes an increased incidence of cardiovascular episodes in patients with DM, raising platelet activity, and mobilizing pro-inflammatory mononuclear cells. Therefore, COVID-19 worsens the glycemic profile in individuals with underlying DM, further weakening the innate immune response and promoting the liberation of pro-inflammatory cytokines (Gazzaz, 2021).

In addition to exacerbated glycemic changes, SARS-CoV-2 also activates complement regulators and complement (Bhaskar et al., 2020), which increases coagulopathy and the cytokine storm, two dangerous complications in severe COVID-19 (Bhaskar et al., 2020). The SARS-CoV-2 invasion triggers a fast innate immune action by leukocytes (e.g., neutrophils and macrophages), mostly by type I IFN. Then, viral particles stimulate a complement cascade *via* the lectin pathway. The complement peptides C3a and C5a are important chemoattractant molecules and promote the migration of neutrophils to the site of infection. The complement membrane attack complex (MAC) causes cell death, releasing damage-associated molecular patterns (DAMPs) (Larenas-Linnemann et al., 2020). If this immune response is ineffective, considerable damage might emerge in capillaries (or other small vessels nearby the alveolar spaces), an event that can activate a pro-coagulant condition. With further virus persistence, complement-initiated damage to vessels increases, and inflammatory cells promote a stronger and wider burst of cytokines, which sustains bidirectional progress of the immune–coagulation axis (Pryzdial et al., 2022).

C3a, and C5a (together with immunoglobulin IgG and C4 consumption) have been found at high levels in patients with COVID-19, elevating according to the severity of the disease (Gao et al., 2020; Marcos-Jiménez et al., 2020). C5a has a strong chemotactic action, influencing the formation of NETs (de Bont et al., 2019) and the migration of neutrophils (Ehrengruber et al., 1994).

Complex type serine proteases are extended by independent additional areas primarily at the N-terminus and interact with many other proteins in complex patterns. In this sense, typical representatives are clotting components and elements of the complement cascade (Pryzdial et al., 2022). Interestingly, the acceleration of the clotting process and clot formation in whole blood and platelet-poor plasma are physiological events that can be noted in the pathophysiology of COVID-19 (Parato et al., 2021).

NE is a serine protease stored within the primary granules of neutrophils. Both the activity and level of NE are higher in blood samples from patients with severe COVID-19 with ARDS (Guéant et al., 2021). After being liberated into plasma, NE is quickly inactivated by endogenous protease inhibitors. *In vitro* analysis demonstrates that sera from COVID-19 patients restrain the activity of exogenous NE, indicating that the NE in the blood of patients with COVID-19 presents a degree of resistance to its endogenous inhibitors (Leppkes et al., 2020).

The plasma kallikrein–kinin system (KKS) includes a group of plasma proteins that respond to tissue damage and pathophysiological stimuli, specifically a non-enzymatic cofactor (high-molecular-weight kininogen) and two serine proteinases (prekallikrein and coagulation factor XII) (Colman and Schmaier, 1997). KKS proteins interact with many pathophysiologic systems, such as the complement and immune systems (Wu, 2015). SARS-CoV-2 disrupt the renin-angiotensin–aldosterone system (RAAS) and KKS, leading to the bradykinin storm, a response associated with increased expression of bradykinin and of its resulting downstream mediated effects. In such a condition, bradykinin is at the center of many important symptoms of COVID-19, such as leaky blood vessels, loss of sense of taste and smell, organs abnormal coagulation, and fluid accumulation in tissues (Rex et al., 2022).

Complement activation also triggers NETs in COVID-19 (Skendros et al., 2020). The sera from COVID-19 patients trigger NET release by healthy control neutrophils *in vitro* (Zhou et al., 2020b), and viable SARS-CoV-2 directly promotes human neutrophils to release NETs in a dose-dependent manner (Veras et al., 2020).

COVID-19 patients who were admitted to the ICU had a greatly elevation in blood neutrophil counts as compared to other SARS-CoV2-positive patients with less severe symptoms (Delgado-Rizo et al., 2017; Zhou et al., 2020a). Besides, an elevation in neutrophils and the neutrophil-to-lymphocyte ratio (NLR) suggests the occurrence of severe or critical diseases with a poor prognosis (Zhang et al., 2020). Nicolai et al. found that patients with COVID-19 have neutrophil–platelet aggregates in blood samples and a different platelet and neutrophil response pattern, which changes with the disease severity (Nicolai et al., 2020).

**Figure 3:**
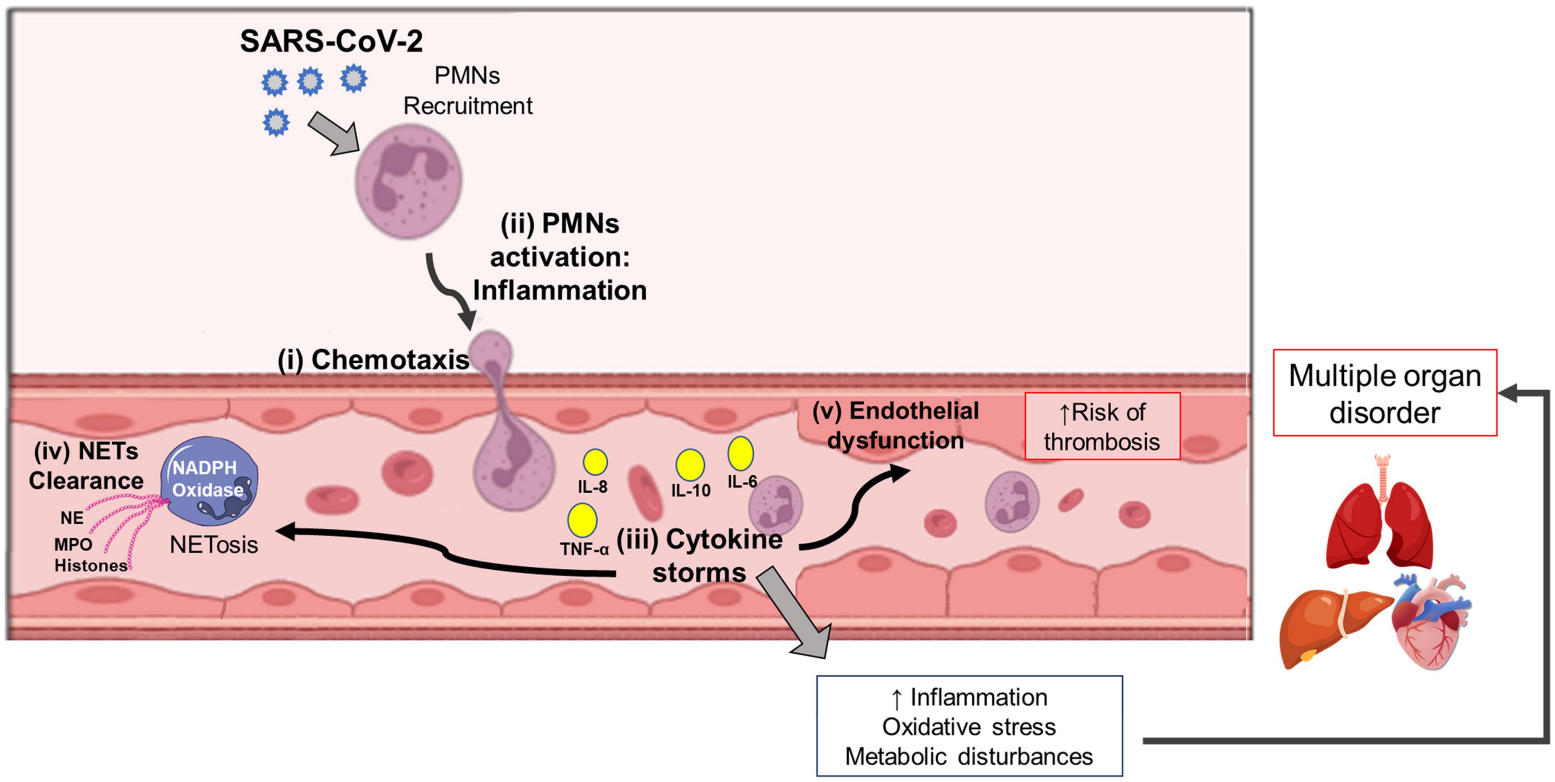
Interplay among Neutrophil, Cytokine Storm, AND Multi-Organ Dysfunction In Covid-19 Patients. Following the host-viral interaction, the SARS-CoV-2 conducts a signaling cascade of crosstalking among the virus recognition mechanism, neutrophil activation, and inflammatory stimuli. After chemotaxis and neutrophil (PMNs) recruitment (i), it occurs cell activation followed by inflammation (ii), and cytokine storm (iii). During these immune events, the NETosis process can protect the host during the virus response by NET clearance (iv). However, exacerbating hyperinflammation in COVID-19 patients is also possible, thus leading to endothelial dysfunction (v), oxidative stress, and metabolic disturbances, which can later result in multiple organ disorders. SARS-CoV-2: severe acute respiratory syndrome coronavirus-2; PMNs, neutrophils; NETs, neutrophil extracellular traps; TNF, tumor necrosis factor; IL, interleukin; NE, neutrophil elastase; MPO, myeloperoxidase.

Middleton et al. (Middleton et al., 2020) showed that plasma MPO-DNA complexes are elevated in COVID-19 patients and that the increased NET formation correlates with COVID-19-related ARDS. These findings indicate the timely application of therapeutic interventions that can disrupt the vicious cycle of COVID-19 immunothrombosis/−thromboinflammation by targeting neutrophil response and NET formation (Figure 3). More in-depth research into the neutrophil response mechanism targeting NETosis in the different phases of COVID-19 is discussed by Borges et al. (2020).

## Concluding remarks

A better understanding of obesity and type 2 diabetes mellitus and severe complications after COVID-19 infection is crucial for the treatment to prevent severe symptoms and complications in these patients. Chronic inflammation and hyperglycemia, specific and usual characteristics of obesity and DM2, contribute to metabolic disturbances in different leukocytes, including neutrophils, lymphocytes, and macrophages, favoring the pro-inflammatory response of these cells. In addition, SARS-CoV-2 replication is favored by metabolic characteristics of these cells in DM2 and obesity condition. Thus, obesity and DM2 are important risk factors for pro-inflammatory response and metabolic dysregulation that can favor the occurrence of the cytokine storm, implicated in the severity and high mortality risk of these patients with COVID-19. At the present, there are limited and few detailed studies about the metabolic changes in leukocytes of obese and type 2 diabetic patients during COVID-19. Additional works addressing the modulation of metabolic pathways are required for the comprehension of the mechanisms involved in this process. Therefore, this better comprehension will be fundamental to direct further studies for the investigation and identification of potential molecular and metabolic targets for the prevention and/or treatment of COVID-19 in obese type 2 diabetic patients, aiming to reduce the pro-inflammatory response and metabolic disturbances associated with the worse prognosis in these individuals.

## Author contributions

SH, RC, TP-C, RG, and LM conceived the study. TL, MG-F, JP, IC, BS, BD, JO, CS, ES, MA, LF, SP, TS, LB, SH, RG, LM, EH, RC, and elaborated the figures and tables, made the literature review and wrote the manuscript. AL-P, MC-B, MV assisted the writing and revision of the manuscript. All authors contributed to the article and approved the submitted version.

## Funding

The authors of this study are supported by grants from the São Paulo Research Foundation (FAPESP, São Paulo, SP, Brazil; 2018/09868–7, 2018/07283–1 and 2021/00200–6), the Coordination for the Improvement of Higher Education Personnel (CAPES, Brasilia, Brazil), the National Council for Scientific and Technological Development (CNPq, Brasilia, Brazil) and the Pro-Rectory of Post-Graduate and Research of the Cruzeiro do Sul University (PRPGP/Cruzeiro do Sul, São Paulo, SP, Brazil).

## Conflict of interest

The authors declare that the research was conducted in the absence of any commercial or financial relationships that could be construed as a potential conflict of interest.

## Publisher’s note

All claims expressed in this article are solely those of the authors and do not necessarily represent those of their affiliated organizations, or those of the publisher, the editors and the reviewers. Any product that may be evaluated in this article, or claim that may be made by its manufacturer, is not guaranteed or endorsed by the publisher.
